# A machine learning approach for predicting suicidal ideation in post stroke patients

**DOI:** 10.1038/s41598-022-19828-8

**Published:** 2022-09-23

**Authors:** Seung Il Song, Hyeon Taek Hong, Changwoo Lee, Seung Bo Lee

**Affiliations:** 1 Department Occupational Therapy, Gumi University, Yaeun-ro 37, Gumi, 39213 South Korea; 2 Department Rehabilitation Science, Daegu University, Gyeongsan, South Korea; 3 Office Hospital Information, Seoul National University Hospital, Seoul, South Korea; 4 Department of Medical Informatics, Keimyung University School of Medicine, Dalgubeol-daero 1095, Dalseo-gu, Daegu, 42601 South Korea

**Keywords:** Psychology, Diseases, Health care, Risk factors

## Abstract

Currently, the identification of stroke patients with an increased suicide risk is mainly based on self‐report questionnaires, and this method suffers from a lack of objectivity. This study developed and validated a suicide ideation (SI) prediction model using clinical data and identified SI predictors. Significant variables were selected through traditional statistical analysis based on retrospective data of 385 stroke patients; the data were collected from October 2012 to March 2014. The data were then applied to three boosting models (Xgboost, CatBoost, and LGBM) to identify the comparative and best performing models. Demographic variables that showed significant differences between the two groups were age, onset, type, socioeconomic, and education level. Additionally, functional variables also showed a significant difference with regard to ADL and emotion (p < 0.05). The CatBoost model (0.900) showed higher performance than the other two models; and depression, anxiety, self-efficacy, and rehabilitation motivation were found to have high importance. Negative emotions such as depression and anxiety showed a positive relationship with SI and rehabilitation motivation and self-efficacy displayed an inverse relationship with SI. Machine learning-based SI models could augment SI prevention by helping rehabilitation and medical professionals identify high-risk stroke patients in need of SI prevention intervention.

Stroke is a cerebrovascular disease characterized by neurological deficits, including hemiplegia, sensory dysfunction, aphasia, neglect, and intellectual and mental disabilities^1^. Post-stroke depression (PSD) is considered the most frequent and important sequela of stroke^2^, and is the largest indicator of the occurrence of suicidal ideation (SI)^3^. COVID-19 pandemic may increase the prevalence of psychiatric disorder and suicide rates during and after the pandemic and this increase in suicides can be attributed to fears of contracting the illness, fears of being a burden to the family, anxiety, social isolation and psychological distress^4^. Such mental health issues may increase SI risk, especially in patients with PSD^5,6^.

SI precedes suicidal attempts or suicidal behaviors, and understanding the effect of SI contributes to understanding and preventing the risk of suicidal behavior^7^. SI is more prevalent among those with persistent physical and cognitive impairments resulting from stroke^8^. The prevalence of suicidal ideation among stroke patients was 13.99%^9^. In this way suffering a stroke was significantly associated with suicidal ideation^10^. In other words, given the high prevalence of suicidal ideation in stroke patients, there is a need to evaluation related factors and performance thorough screenings in this population^9^. Previous studies have reported that the occurrence of depression and mood disorders increases SI in stroke patients, and that there is a significant positive correlation between depression and SI in stroke patients^11,12^. Therefore, a clinical data prediction model is necessary to reduce SI in patients after a stroke.

Most of the developed stroke prediction models are reported in studies on diagnosis, sequela, mortality, and physical function, and cannot be conveniently used practically owing to the associated invasive measurements and analyses^13–16^. Additionally, while studies on predictive model development for stroke-related emotional disorders, such as post-stroke anxiety and PSD have been conducted^17,18^, the predictors used in these models were assessed at one-month post-stroke, at which point full depressive symptoms may not be present. Additionally, procedures need to be devised for the comparison of different machine learning models to select the best among them.

This study presents a stroke patient SI prediction model independent of biochemical data that are not routinely collected and aims to differentiate SI. For this purpose, we used the data collected from a specialized hospital in Daegu Metropolitan City, Republic of Korea, to predict high or low levels of SI outcomes in patients with stroke. To date, there have been no similar studies, and most of the developed models require image data and invasive test data, which are difficult to collect. This study is also the first to apply the best model selected after comparing the performance of three boosting models using medical history, demographic and psychological factors, cognitive and activities of daily living (ADL) function data collected from a sample of subacute and chronic stroke patients in an attempt to create an SI prediction tool.

## Methods

### Setting, data description, and pre-processing

A total of 385 stroke patients were screened for eligibility between October 2012 and March 2014. The eligibility criteria were as follows: diagnoses confirmed based on the results of magnetic resonance imaging and computed tomography images evaluated by a physician; patients in the age range of 18–80 years; a diagnosis of ischemic and hemorrhage stroke type; and patients with an onset of subacute stroke between one and six months and chronic stroke over six months. The collected anonymized sample data included information on demographics, hospital admission, cognitive function, motor function, ADL, and emotion assessment results. The ethics committee of our Institutional Review Board reviewed this study. This is a retrospective study using anonymized data obtained with written consent from all patients. This study has been the ethics committee of Daegu University Institutional Review Board (IRB) approved this study (1040621-202111-HR-079) and all methods were performed in accordance with the relevant guidelines and regulations.

The features obtained from pre-processing were then divided into five domains based on the assessment for which they were collected. All the potential predictors, including sociodemographic factors, cognitive function, motor function, ADL, and emotional parameters, were extracted from the hospital’s electronic medical records and experimental data. Assessments included the Scale for SI^19–21^, the Korean version of the Mini-Mental State Examination (MMSE-K)^22^, the Manual Function Test (MFT)^23^, the Korean version of the Modified Bathel Index (K-MBI)^24^, Self-Efficacy Scale^25^, the Rehabilitation Motivation Scale (RMS)^26^, the Beck Anxiety Inventory (BAI)^27^, the Beck Depression Inventory (BDI)^28^. The study data indicated that the assessment outcome had high reliability and validity.

Demographic features included sex, age, phase, type, affected side, dominant hand, socioeconomic level, marital status, hypertension, diabetes, family/past history, smoking and drinking, education, and transfer. Cognitive function was measured using the MMSE-K, motor function using the MFT, and ADL using the K-MBI. Finally, positive emotions were measured using the Self-Efficacy scale and the RMS, and negative emotions were measured using the BAI and BDI.

Variables for demographic features, cognitive function, motor function, and ADL, as well as numerical variables for emotion were included in the dataset. The target variable was the SI Scale score. To transform the problem into a binary classification one and to compare our results directly with those obtained by existing methods, we discretized the SI into two classes: high SI group ($>$ 14) and low SI group ($\le$ 14)^19–21^. This particular discretization is medically relevant because it helps to distinguish between stroke patients who will be able to live an independent life from those with a significant suicide risk.

**Figure 1:**
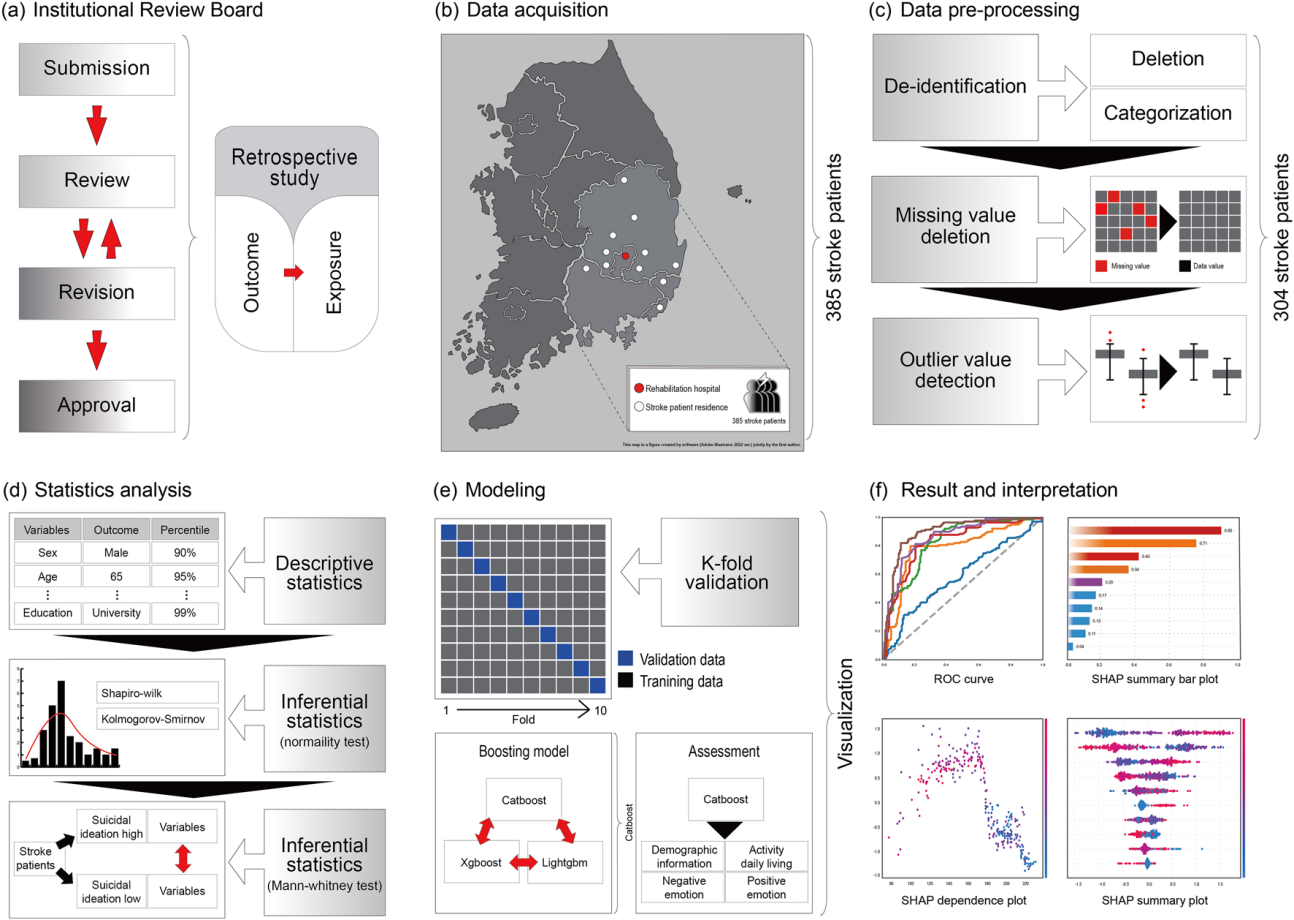
Stroke suicidal ideation prediction model.

The age variables were transformed into categorical variables. Two pre-processing methods were used to eliminate the outliers and missing values. For patient data containing missing values, the deletion technique was used^29^. Outliers were selected as results outside the upper and lower limits based on the quartile and were deleted^30^. After data cleaning, the resulting dataset contained 23 features, and the data of 304 patients who met the inclusion criteria were included in the datasets, which were then used for model training and validation (Fig. 1). All the stroke patients included in the study were screened, and anonymized data were used for a retrospective study comprising two groups: high SI group (n = 165) and low SI group (n = 139).

### Statistical analysis

The data were analyzed using the IBM Statistical Package for Social Sciences (SPSS) version 25.0. Frequency analysis and chi-square test were performed, and a normality test was performed to determine normality of the distributions. The age variable was collected into a categorical variable for anonymization. The study data does not contain a continuous age variable. However, it does have a categorical age variable, which is composed of multiple age groups of varying widths^31^; it was converted into a 10-year interval based on the original data. Two groups (high and low SI) were divided based on a score of 14 based on cut-off points in three SI studies^19–21^ and consultations with two psychiatric and rehabilitation experts. And the Mann–Whitney U (two tailed) test was conducted to determine statistically significant difference in the variables (demographic information, cognitive, motor, ADL, emotional function) between the two groups. Differences were considered statistically significant at p < 0.05 (Fig. 1). Three models (Xgboost, CatBoost, LGBM) were compared and the one with the best performance, that is the CatBoost model, was selected.

### SI prediction model

We used an ML approach to develop the SI prediction models for stroke patients. The three boosting models (Xgboost, CatBoost, and Light GBM [gradient booting model]) apply an algorithm based on gradient boosted decision trees. Xgboost implements the gradient boosting algorithm, which combines numerous decision trees for elaborate classification, in a fast and generalized manner^32^. XGBoost also applies a sparsity-aware algorithm to find the best split faster than the other methods. Light GBM (LGBM) is an advanced implementation of gradient boosting. This algorithm differs from the other algorithms in the growth of the tree in-depth or by leaves. LGBM handles large amounts of data with the lowest memory requirements^33,34^. Almost all the modern gradient-based methods work well with numerical attributes. If the dataset contains both numerical and categorical variables, then the categorical ones must be converted to numerical ones; this however leads to a potential decrease in the model’s accuracy. CatBoost is a gradient enhancement library whose main advantage lies in that it works well with categorical features^35^. One-hot encoding is used for processing categorical features, but this method incurs more computational complexity and memory owing to its high cardinalities. Therefore, an effective way to process categorical features is to use the CatBoost algorithm based on modified target statistics.

### Model performance evaluation

In the previous section, the variables that showed a significant difference between the two groups were selected through a traditional statistical analysis. The stroke SI model was tested using the ten-fold cross validation dataset^36^. The values of the hyperparameters were optimized and the optimization was performed and the tested values (Supplementary 1). The overall model predictive performance was assessed using the area under the receiver operating characteristic (ROC) curve. The performance characteristics of the stroke SI model indicate sensitivity, specificity, accuracy, positive predictive value (PPV), and negative predictive value (NPV) results. A sub-assessment was performed by selecting the model with the highest performance. For each assessment, a separate ROC curve was generated using the predictions obtained from the highest-performance model and the outcomes within each assessment. The importance and relationship of stroke SI variables were derived through Shapley additive explanation (SHAP) values. The red and blue dots indicate that the variables at that point had positive and negative effects on the SI occurrence, respectively. The data were analyzed using Python 3.7.12 (Python Software Foundation).

### Ethics approval and consent to participate

The ethics committee of Daegu University Institutional Review Board (IRB) approved this study (1040621-202111-HR-079). This is a retrospective study using anonymized data obtained with written informed consent from all patients. This study has been independently reviewed and approved by an IRB.

## Results

The demographic data of the stroke patients are presented in Table 1. The variables that showed a significant difference between the two groups based on the SI outcome criterion were age, onset, type, socioeconomic level, and education level (*p* < 0.05). The high SI group had a higher frequency of older adults over 65 years of age than the low SI group. The onset group had a higher frequency of older adults when the stroke onset was less than 6 months, the socioeconomic level was poor, and the education level was low.

**Table 1:** Demographic and clinical characteristics based on suicidal ideation.

Variables	Total (*n* = 304)		SI low (*n* = 165)		SI High (*n* = 139)		
	n	%	n	%	n	%	*p*
**Sex**							.090
Male	220	72.4	126	76.4	94	67.6	
Female	84	27.6	39	23.6	45	32.4	
**Age**							.016
Under 45	76	25.0	49	29.7	27	19.4	
45–54	131	43.1	67	40.6	64	46.0	
55–64	66	21.7	39	23.6	27	19.4	
65 over	31	10.2	10	6.1	21	15.1	
**Onset**							.015
Subacute	174	57.2	84	50.9	90	64.7	
Chronic	130	42.8	81	49.1	49	35.3	
**Type**							.009
Ischemic	235	77.3	137	83.0	98	70.5	
Hemorrhage	69	22.7	28	17.0	41	29.5	
**Affected side**							.084
Right	178	58.6	104	63.0	74	53.2	
Left	126	41.4	61	37.0	65	46.8	
**Dominant hand**							.242
Right	291	95.7	160	97.0	131	94.2	
Left	13	4.3	5	3.0	8	5.8	
**Socioeconomic level**							.001
High (Health insurance)	267	87.8	154	93.3	113	81.3	
Low (Medical care)	37	12.2	11	6.7	26	18.7	
**Hypertension**							.870
Yes	180	59.2	97	58.8	83	59.7	
No	124	40.8	68	41.2	56	40.3	
Diabetes							.295
Yes	90	29.6	53	32.1	37	26.6	
No	214	70.4	112	67.9	102	73.4	
**Marital status**							.056
Married	222	73.0	124	75.2	98	70.5	
Unmarried	59	19.4	34	20.6	25	18.0	
Divorced/widowed	23	7.6	7	4.2	16	11.5	
**Family history**							.092
Yes	170	55.9	85	51.5	85	61.2	
No	134	44.1	80	48.5	54	38.8	
**Past history**							.421
Yes	119	39.1	68	41.2	51	36.7	
No	185	60.9	97	58.8	88	63.3	
**Smoking**							.408
Yes	174	57.2	98	59.4	76	54.7	
No	130	42.8	67	40.6	63	45.3	
**Drinking**							.791
Yes	262	86.2	143	86.7	119	85.6	
No	42	13.8	22	13.3	20	14.4	
**Education**							.017
Uneducated	12	3.9%	1	0.6	11	7.9	
Elementary	7	2.3%	4	2.4	3	2.2	
Middle School	18	5.9%	8	4.8	10	7.2	
High School	216	71.1%	125	75.8	91	65.5	
University	51	16.8%	27	16.4	139	17.3	
**Transfer**							.108
Wheelchair	91	29.9	43	26.1	48	34.5	
Ambulation	213	70.1	122	73.9	91	65.5	

Abbreviation: SI, suicidal ideation.

The results presented in Table 2 indicate a significant difference in ADL and emotions in both the groups (*p* < 0.05). In particular, there was a significant difference between the two groups in the emotional domain (*p* < 0.001). Cognition and motor functions, on the other hand, did not differ between the two groups.

**Table 2:** Comparison of cognitive functions, motor functions, ADL, emotional functions between both groups.

Domain	SI low (*n* = 165)			SI High (*n* = 139)		
	Mean (*SD*)	IQR		Mean (*SD*)	IQR	*p*
**Cognition**						
MMSE	23.99 (1.54)		23–25	23.91 (1.70)	23–25	.784
Motor						
MFT	22.28 (2.04)		21–24	22.03 (1.78)	21–23	.207
**ADL**						
MBI	72.45 (5.99)		69–77	71.00 (6.59)	66–77	.023
**Emotion**						
Self-efficacy	189.18 (30.61)		181–216	154.24 (30.89)	131–156	.001
Rehabilitation motivation	90.77 (14.61)		81–100	73.14 (14.37)	63–82	.001
BAI	15.53 (4.04)		12–17	21.83 (5.22)	18–23	.001
BDI	14.35 (4.47)		13–18	20.08 (4.62)	19–24	.001

Abbreviations: SI, suicidal ideation; *SD*, standard deviation; IQR, interquartile range; MMSE, mini-mental state examination; MFT, manual function test; MBI, modified Bathel index; BAI, beck anxiety inventory; BDI, beck depression inventory.

Table 3 shows the combined analysis of one evaluation tool indicating a significant difference between the two groups and all demographic information variables indicating a significant difference between the two groups. As shown in Table 3, emotional features such as BDI (depression), BAI (anxiety), self-efficacy, and rehabilitation motivation showed generally better results than MBI in the CatBoost model. Sensitivity and NPV were rehabilitation motivation, specificity was MBI, and PPV was self-efficacy, with BDI having the highest accuracy. Additionally, as for the cut-off points, BDI showed a mild depressive state, and MBI showed a cut-off point of moderate dependence, whereas BAI showed a normal level cut-off point. Supplementary information 2 shows the measure value analyzed by combining the demographic information and the entire evaluation tool that showed a significant difference between the two groups. Among the three models, the area under the AUC value was higher for the CatBoost model than the other two models, and most values (sensitivity, NPV) outperformed the XGBoost and LGBM scores (Supplementary information 2).

**Table 3:** Result of the CatBoost model based on emotion and ADL data.

Variables	Sensitivity	Specificity	PPV	NPV	Accuracy	Cut off value
MBI	.317	.861	.656	.599	.612	68
Self-efficacy	.799	.824	.792	.829	.812	177
Rehabilitation motivation	.899	.636	.675	.882	.757	86
BAI	.791	.794	.763	.818	.793	18
BDI	.813	.794	.768	.834	.803	19

Abbreviations: ADL, activity daily living; PPV, positive predict value; NPV, negative predict value; MBI, modified bathel index; BAI, beck anxiety inventory; BDI, beck depression inventory.

Supplementary information 3 shows the ROC curve of the analysis results in Table 3 and the ROC curve analyzed by combining the demographic information and the entire evaluation tool that showed a significant difference between the two groups. Supplementary information 3 shows the ROC curves of the CatBoost classifier for the five functional assessments. The AUC values were ordered as per the order presented in Table 3: first, negative emotion evaluation, such as evaluation of depression and anxiety; second, positive emotion evaluation; and third, ADL assessment. Furthermore, the AUC value, which includes the demographic variables that indicated a significant difference between the two groups, as well as the exercise and emotion evaluation, showed the highest result.

**Figure 2:**
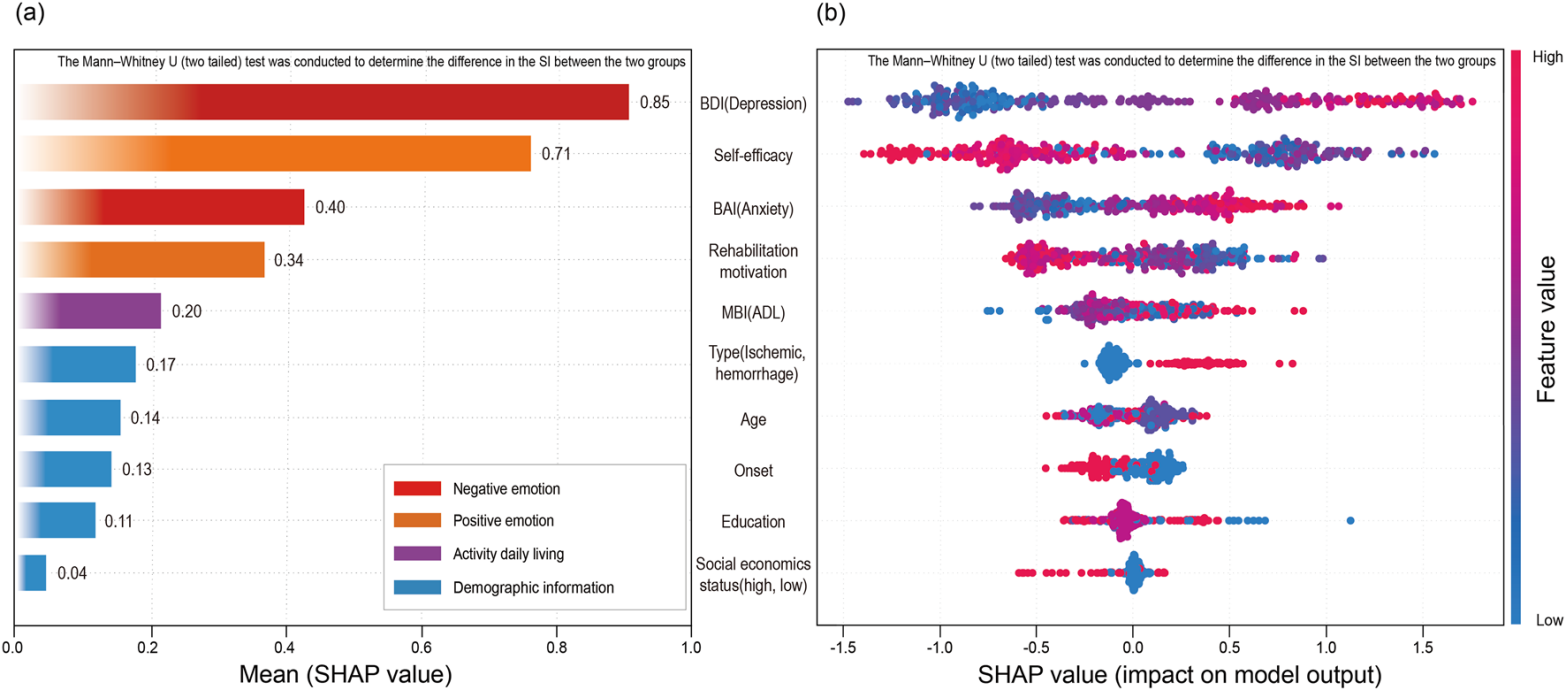
Feature importance based on SHAP values (The red and blue dots indicate that the variables at that point had positive and negative effects on the SI occurrence, respectively): (**a**) Mean absolute SHAP values (**b**) Summary.

Regarding SHAP, depression was found to be the most important predictor for SI in stroke patients, followed by emotional variables such as self-efficacy, anxiety, and rehabilitation motivation. In the SHAP summary plot result (Fig. 2), it was seen that the higher the negative emotions such as depression and anxiety, the higher the SI. Conversely, the lower the positive emotions such as self-efficacy and rehabilitation motivation, the higher the SI.

**Figure 3:**
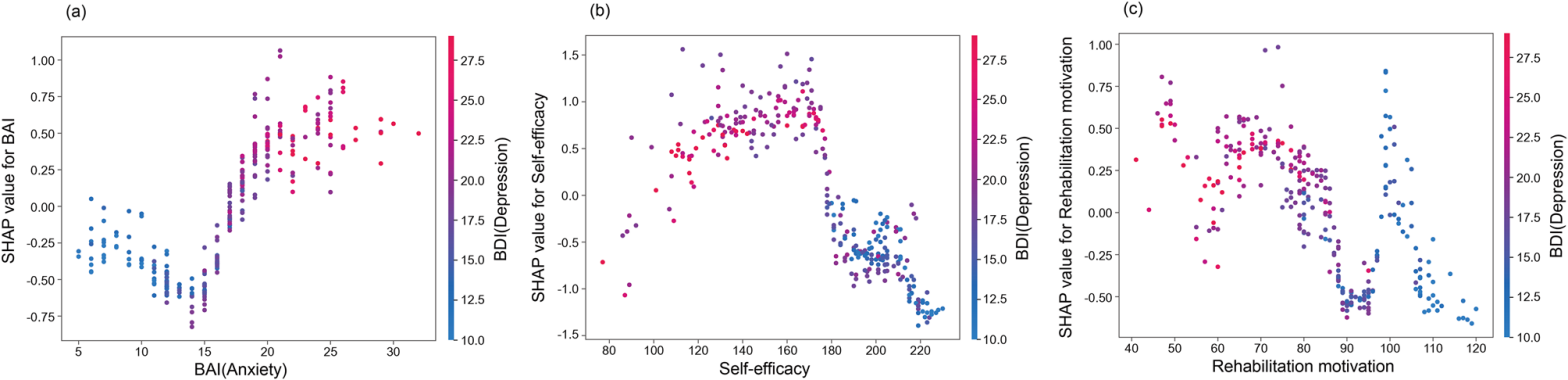
Partial dependence plot by SHAP value. Relationship between (**a**) self-efficacy and depression (**b**) rehabilitation motivation and depression (**c**) anxiety and depression.

Using the SHAP dependence plot, the results of the interaction relationship between anxiety, rehabilitation motivation, self-efficacy, and ADL that exhibited significant differences were derived based on depression, which demonstrated the greatest importance for SI in stroke patients. Negative emotions, such as anxiety and depression, showed a positive relationship, and positive emotions, such as rehabilitation motivation and self-efficacy, exhibited an inverse relationship with SI. There was no evident association between depression and ADL function (Fig. 3).

## Discussion

In this study, using stroke patients’ data from a rehabilitation hospital, we developed and validated a model for SI prediction in stroke patients within a post-onset period. Using the statistically significant predictors that a stroke patient can report in a direct interview and survey, performance was compared for the three boosting models.

Using the chi-square test for the demographic variables used in this study, statistically significant differences were observed between the two groups divided on the basis of age, onset, stroke type, and economic and education level. Among them, the high SI group had a high proportion of participants aged 65 years, an onset of less than six months, hemorrhagic stroke, and low economic, and education levels. This suggested that risk factors for SI in stroke patients increased in various pathologies due to rapid changes that take place associated with old age, loss and maladaptation immediately after onset^11^, hemorrhagic stroke, severe pain, poor prognosis^37^, low socioeconomic level, and low educational level. This can be seen as a low-income group^38,39^. Additionally, there was a significant difference for widowed or divorced patients, which showed an approximate result (Table 1). This finding was consistent with a previous study that indicated a large difference depending on whether the support of the family or spouse was present^40^.

Based on the study results, a statistically significant difference between the two groups in the variables of ADL and emotional function was noted. In previous studies, cognitive dysfunction was found to be associated with suicide^39,41^, which was not observed in the results of the current study. The cognitive function evaluation tool used in this study, the MMSE, is simple and efficient; however, we believe that it may have been affected by low sensitivity, as it is a screening tool for mild cognitive impairment^42^. In the case of MFT, lower extremity functions, such as gait function^43,44^, that affect depression in stroke patients were not included, and so, there was no significant difference between the two groups. In contrast, depression can be viewed as the biggest risk factor for SI according to previous studies’ results^43^, and has previously showed a strong correlation with ADL, anxiety, self-efficacy, and Rehabilitation motivation^45,46^. Therefore, it is thought that there was a significant difference between the two groups in ADL and emotional variables.

Only statistically significant demographic and functional domain variables were applied to the three boosting models to derive their respective performances^47,48^. After comparing the performance of the three models, it was found that LGBM had the most inferior performance, whereas Xgboost showed the best performance in terms of specificity, PPV, and accuracy. Further, CatBoost showed the best performance in terms of sensitivity, NPV, and AUC (Supplementary information 2). While XGBoost and LightGBM offer several advantages, it must be noted that 16 out of the 23 variables of the stroke data used in this study were categorical. When a large number of categorical features are present in the dataset, then CatBoost may offer a more efficient performance^49^. In addition, LGBM is disadvantageous in that its application to small datasets (i.e., fewer than 10,000 cases) leads to leaf-wise growth, which, in turn, causes significant overfitting, whereas XGBoost cannot handle categorical features on its own^50,51^. Additionally, the classification performance improved when more features were added to the classifiers (Supplementary information 3). The predicted results can be used to take the necessary precautions and improve the function of stroke patients. Further, the AUC of the best classifiers was approximately 0.900. This value can be said to be sufficient for the reliable prediction of patients’ functional outcomes^52^.

Figure 2a shows the absolute influence of each variable of CatBoost through SHAP on the model. Notably, it is crucial for physicians to understand the effect of various factors on the SI of stroke patients. The variable that showed the greatest influence on stroke occurrence in patient SI was “depression,” followed by “self-efficacy,” “anxiety,” “rehabilitation motivation,” and so forth. The emotion function level had a significant influence on the occurrence of SI in stroke patients. Figure 2b is a SHAP summary showing the degree of influence of each variable on stroke patient SI prediction. Thus, higher levels of “depression” and “anxiety” meant that the probability of SI occurrence increased^53^. Therefore, the higher the “self-efficacy” and “rehabilitation motivation,” the lower the probability of SI occurrence, thereby exhibiting an inverse relationship with each other. Figure 3 is a SHAP partial dependent plot showing the correlation between depression, the most influential SI predictor in stroke patients, and other important predictive factors. Positive emotions, such as rehabilitation motivation, and self-efficacy, are observed to have a negative correlation (Fig. 3 b, c). The results thus obtained were identical to those reported in previous studies on depression, anxiety, rehabilitation motivation, and self-efficacy in stroke patients; negative and positive emotions were found to be the main factors affecting the SI of stroke patients; further, it was found that the two had opposite effects on each other^54–56^.

The stroke SI prediction model developed in this study can therefore be used to classify stroke patients into low- and high-risk SI groups based on routinely collected medical data and self-report questions. Furthermore, improved characterization of low and high risk for stroke-related SI can be achieved by analyzing the importance and correlation of the model’s prediction features. The implementation of a stroke SI prediction model in public health systems may facilitate early stroke SI detection and intervention programs, thereby reducing suicidal ideation. Additionally, it should be noted that a prediction model is only a tool to support the clinician and therefore cannot be used to replace personal judgment.

### Limitations

This study has some limitations. First, prospective clinical trials are needed to demonstrate a clear clinical benefit of the addition of a stroke SI prediction model to the clinical intervention system. Clearer information about risk predictors can be provided by collecting additional data. Second, the study results cannot be generalized for all stroke features, such as biochemical indices and lesion location, which are also considered risk factors. Future studies should combine these to reveal the interactions of pathophysiological risk factors^17^. In a follow-up study, the model may benefit from the inclusion of as yet unavailable contributing predictors, such as invasive test data like quantitative brain structural and functional imaging data of stroke patients.

## Conclusion

We constructed a comprehensive risk prediction model for SI in stroke patients based on clinical and psychological features. The model indicated that psychological factors were important for identifying SI risk in subacute and chronic stroke patients and contributed to post-stroke rehabilitation and mental health. Furthermore, the prediction model ultimately works as a decision tool to help clinicians identify the SI risk early, which will allow the optimization of stroke patients’ suicide prevention strategies in personalized medicine.

## Supplementary Information

Supplementary Information 1.

Supplementary Information 2.

Supplementary Information 3.

## Data Availability

Due to privacy/ethical restriction, data are available from the corresponding author on reasonable request.

## Abbreviations

PSD: Post-stroke depression
SI: Suicidal ideation
ADL: Activities of daily living
MMSE-K: Mini-mental state examination-Korea
MFT: Manual function test
RMS: Rehabilitation motivation scale
K-MBI: Modified bathel index
BAI: Beck anxiety inventory
BDI: Beck depression inventory
LGBM: Light GBM
PPV: Positive predictive value
NPV: Negative predictive value
ROC: Receiver operating characteristic
SHAP: Shapley additive explanation
