# Supplementary material for: A machine learning approach for predicting suicidal ideation in post stroke patients
Source: Sci Rep. 2022 Sep 23;12:15906. doi: 10.1038/s41598-022-19828-8 (PMC9508242; doi:10.1038/s41598-022-19828-8)
Supplement: Supplementary file 1 — Supplementary Information 1. [file 41598_2022_19828_MOESM1_ESM.pdf]

Supplementary information 1. Hyperparameters for each algorithm in the grid search.

Classifier	Tested values	Selected values
Xgboost	n_estimators: 10, 25, 50, 100, 250, 500, 1000<br>learning_rate: 0.001, 0.01, 0.1, 0.2, 0.5<br>max_depth: 2, 3, 5, 10, 25, 50, 100	n_estimators: 500<br>learning_rate: 0.01<br>max_depth: 3
CatBoost	n_estimators: 10, 25, 50, 100, 250, 500, 1000<br>learning_rate: 0.001, 0.01, 0.1, 0.2, 0.5<br>max_depth: 2, 3, 5, 10, 25, 50, 100	n_estimators: 1000<br>learning_rate: 0.01<br>max_depth: 10
LGBM	n_estimators: 10, 25, 50, 100, 250, 500, 1000<br>learning_rate: 0.001, 0.01, 0.1, 0.2, 0.5<br>max_depth: 2, 3, 5, 10, 25, 50, 100	n_estimators: 250<br>learning_rate: 0.1<br>max_depth: 5

Abbreviations: LGBM, light gradient boosting model
