# Supplementary material for: A machine learning approach for predicting suicidal ideation in post stroke patients
Source: Sci Rep. 2022 Sep 23;12:15906. doi: 10.1038/s41598-022-19828-8 (PMC9508242; doi:10.1038/s41598-022-19828-8)
Supplement: Supplementary file 2 — Supplementary Information 2. [file 41598_2022_19828_MOESM2_ESM.pdf]

Supplementary information 1.

Table 1. Comparison of machine learning model values (Three boosting model and logistic regression model)

Classifier	Sensitivity	Specificity	PPV	NPV	Accuracy	AUC
Xgboost	.813	.836	.807	.842	.826	.895
CatBoost	.921	.739	.749	.917	.822	.900
LGBM	.914	.746	.752	.911	.822	.897
LR	.784	.838	.838	.828	.832	.852

Abbreviations: PPV, positive predict value; NPV, negative predict value; AUC, area under the receiver operating characteristics curve; LGBM, light gradient boosting model; LR, logistic regression
