# Supplementary material for: A machine learning approach for predicting suicidal ideation in post stroke patients
Source: Sci Rep. 2022 Sep 23;12:15906. doi: 10.1038/s41598-022-19828-8 (PMC9508242; doi:10.1038/s41598-022-19828-8)
Supplement: Supplementary file 3 — Supplementary Information 3. [file 41598_2022_19828_MOESM3_ESM.pdf]

Supplementary information 2.

Figure 1. Xgboost model ROC curve graph

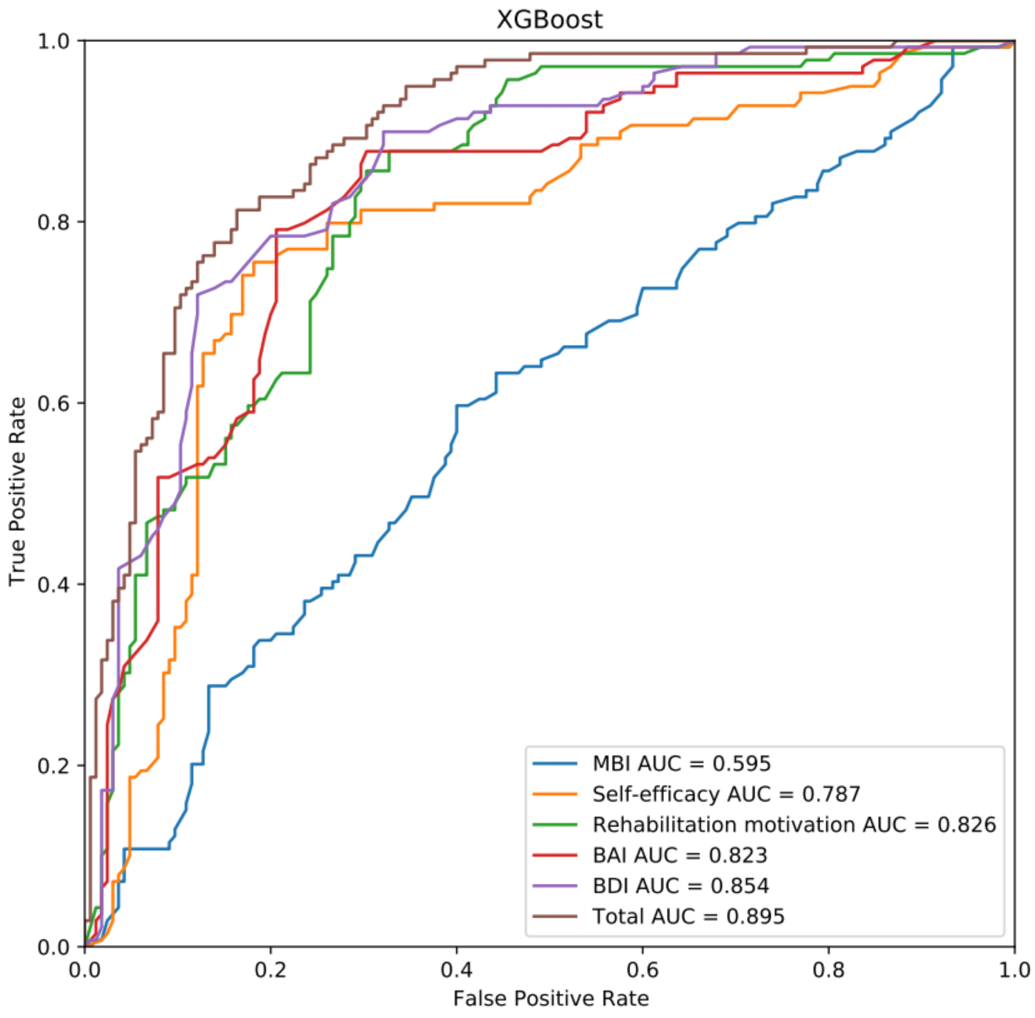

Abbreviations: MBI, modified bathel index; BAI,beck anxiety inventory; BDI, beck depression inventory

Supplementary information 2.

Figure 2. CatBoost model ROC curve graph

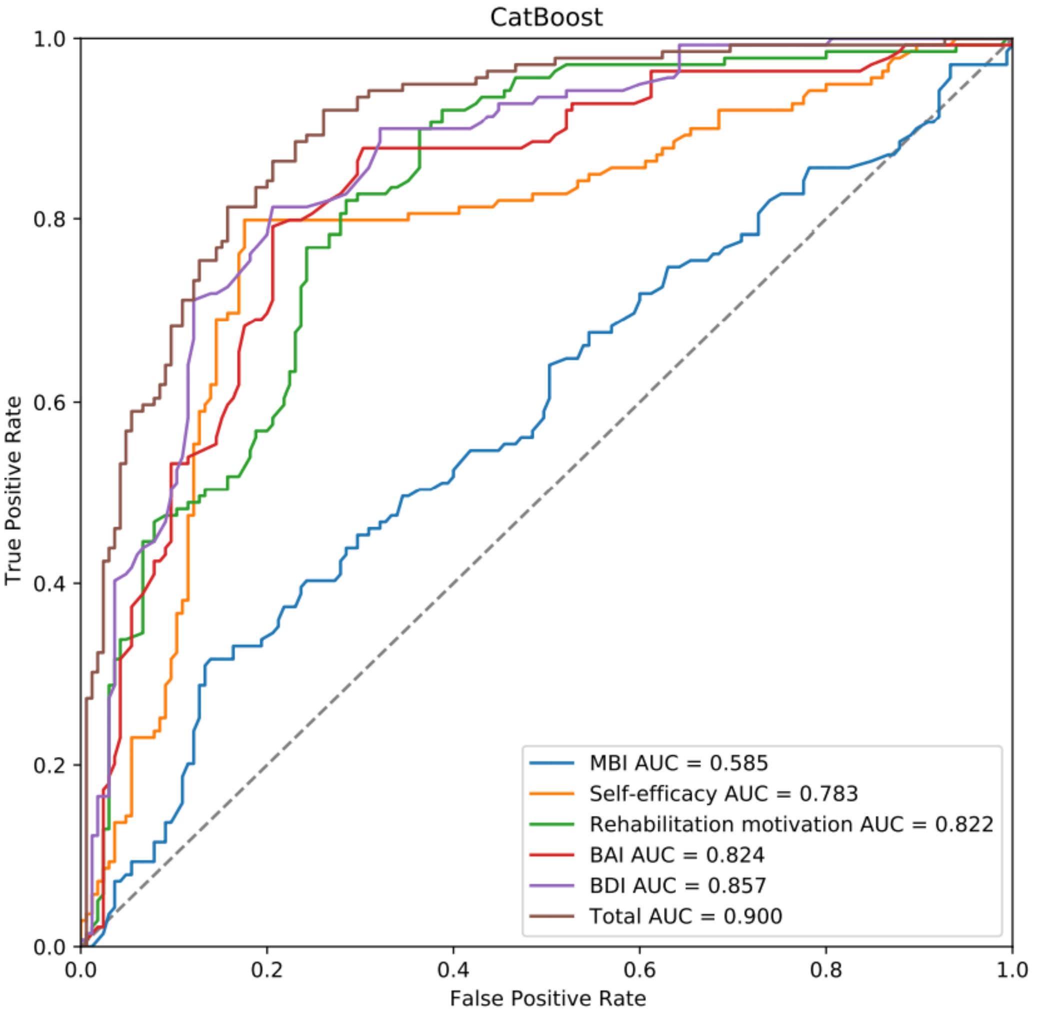

Abbreviations: MBI, modified bathel index; BAI,beck anxiety inventory; BDI, beck depression inventory

Supplementary information 2.

Figure 3. Light GBM (LGBM) model ROC curve graph

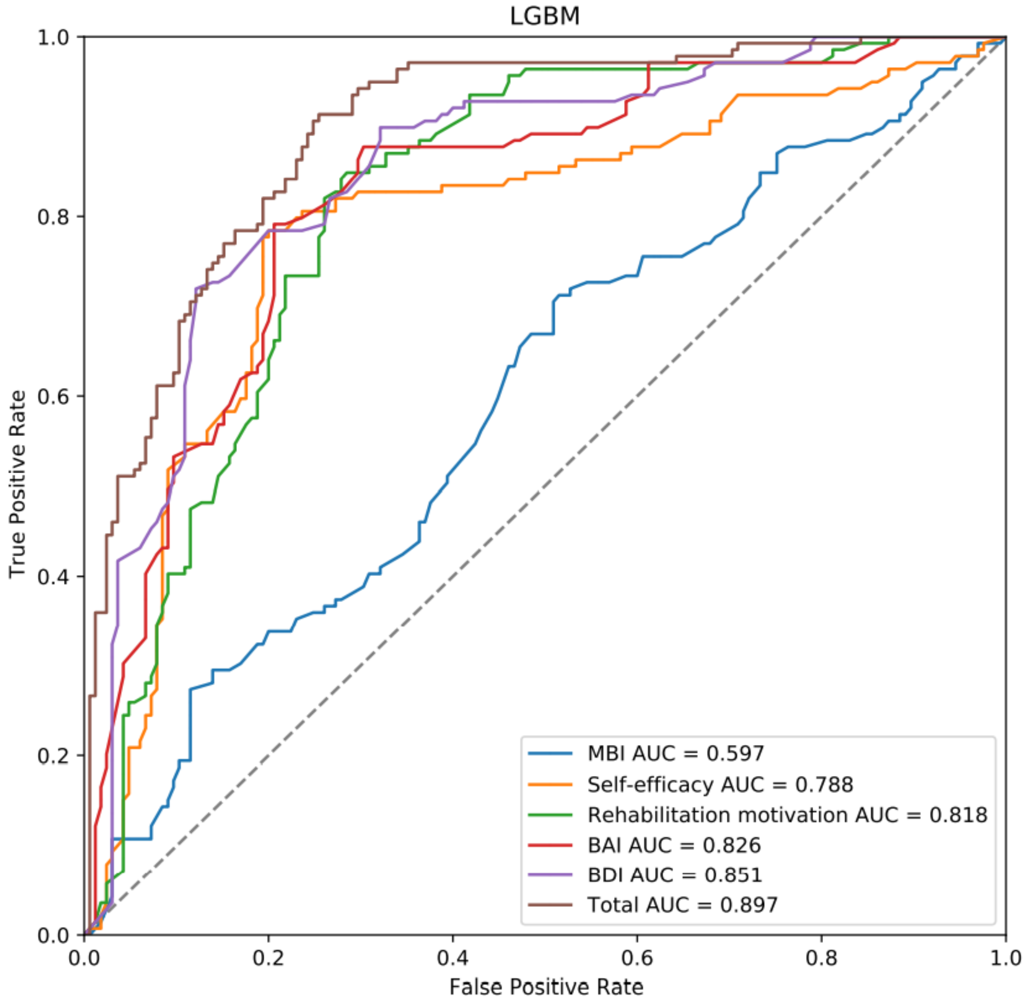

Abbreviations: MBI, modified bathel index; BAI,beck anxiety inventory; BDI, beck depression inventory
